# The Dutch health insurance reform: switching between insurers, a comparison between the general population and the chronically ill and disabled

**DOI:** 10.1186/1472-6963-8-58

**Published:** 2008-03-19

**Authors:** Judith D de Jong, Atie van den Brink-Muinen, Peter P Groenewegen

**Affiliations:** 1 NIVEL-Netherlands Institute for Health Services Research, PO Box 1568, 3500 BN Utrecht, The Netherlands; 2 Utrecht University, Department of Sociology and Department of Human Geography, Utrecht, the Netherlands

## Abstract

**Background:**

On 1 January 2006 a number of far-reaching changes in the Dutch health insurance system came into effect. In the new system of managed competition consumer mobility plays an important role. Consumers are free to change their insurer and insurance plan every year. The idea is that consumers who are not satisfied with the premium or quality of care provided will opt for a different insurer. This would force insurers to strive for good prices and quality of care. Internationally, the Dutch changes are under the attention of both policy makers and researchers. Questions answered in this article relate to switching behaviour, reasons for switching, and differences between population categories.

**Methods:**

Postal questionnaires were sent to 1516 members of the Dutch Health Care Consumer Panel and to 3757 members of the National Panel of the Chronically ill and Disabled (NPCD) in April 2006. The questionnaire was returned by 1198 members of the Consumer Panel (response 79%) and by 3211 members of the NPCD (response 86%). Among other things, questions were asked about choices for a health insurer and insurance plan and the reasons for this choice.

**Results:**

Young and healthy people switch insurer more often than elderly or people in bad health. The chronically ill and disabled do not switch less often than the general population when both populations are comparable on age, sex and education.

For the general population, premium is more important than content, while the chronically ill and disabled value content of the insurance package as well. However, quality of care is not important for either group as a reason for switching.

**Conclusion:**

There is increased mobility in the new system for both the general population and the chronically ill and disabled. This however is not based on quality of care. If reasons for switching are unrelated to the quality of care, it is hard to believe that switching influences the quality of care. As yet there are no signs of barriers to switch insurer for the chronically ill and disabled. This however could change in the future and it is therefore important to monitor changes.

## Background

On 1 January 2006 a number of far-reaching changes in the Dutch health insurance system came into effect. There is now one type of health care insurance for all, where there used to be public insurance for approximately 60% of the population and private insurance for the other 40%. The basic package is compulsory for everyone who lives in the Netherlands or pays wage tax in the Netherlands. Insurance companies are not allowed to select favourable risks or to differentiate the premium according to (proxies for) risk. On a macro level, insurance premiums are half based on nominal premiums and for the other half income dependent [1]. In the new system of regulated competition switching between health insurance companies or policies plays an important role. Insured persons are free to change their insurer and insurance plan every year. Insurers must accept every applicant for the basic package. In the old system of health insurance, people could also switch insurance company. However, the situation was different for the publicly insured and the privately insured. The publicly insured could switch, the nominal premium was much lower and there was less accessible information on premium and service level of insurance companies. The privately insured could also switch, but as a result of risk selection and premium differentiation many were actually locked in with their insurance company.

Internationally, the Dutch changes are under the attention of both policy makers and researchers. The Dutch health insurance reform is part of a broader transformation towards a regulated market for health care. Specific information on the new Dutch health insurance system is given in Table 1. Competition between health insurance companies is not unique for the Netherlands. Other countries with competing health insurers are for instance Germany, Switzerland, Belgium and the USA [2-5]. Introducing more choice in health care is a general trend in western countries [6,7]. The policy assumption is that greater choice of health plans improves quality and lowers costs [8]. Insured can both switch to another health plans from the same insurer, and to another insurer. The focus of this article will be on switching to another insurer. Switching between health insurers and the threat that people can do so, is supposed to induce insurers to adapt their offers to the preferences of their insured. However, it can be discussed what the actual impact of enhanced choice will be, due to legal and non-legal restrictions to consumer choice [9].

**Table 1:** The Dutch health insurance system after the insurance reform of 1 January 2006

**Health care insurance law**	• Introduced on 1 January 2006
	• Abolition of distinction between private and public insurance
	• Insurance under private law with public limiting conditions
	• Obligation for every citizen to take health insurance
	• Risk adjustment
**Insurance policy**	• Free choice between insurance organisations
	• Basic package (identical for everybody)
	• Choice between in-kind and restitution policy
	• Additional insurance (no obligation to accept, not necessarily with same insurer as basic package)
	• Choice of deductible (min. €100, max. €500)
	• No-claim premium restitution
	• Collectives (via work or other) get premium reduction up to 10%

The extent to which people indeed exert their freedom of choice is first of all important from the point of view of the assumptions behind the reforms. Assumptions are e.g. that people indeed want to have more freedom of choice concerning their health insurance company or policy and that they choose on the basis of parameters that are relevant to improve the cost – quality balance. Secondly, the extent to which people actually use the possibility to switch between insurers is important from the point of view of unintended consequences of introducing more choice. If some categories of people have more difficulty in exerting their freedom of choice, they might forego the fruits of system reform and new inequalities might arise. The Dutch health insurance reform was explicitly designed to prevent the development of such inequalities through a standard basic insurance package, the ban on risk selection, and by stimulating the availability of transparent information. However, according to market transition theory in general, transitions of planned economies to markets bring about new inequalities [10,11]. There will be (groups of) people who will benefit from it more than others.

Switching between insurers is an important pillar in the new system. The international literature shows that the numbers of people switching health insurance are usually low. The actual level depends on the availability of choice options, market structure and institutional features, the benefits of switching, and individual inclination, related e.g. to ties to the current insurance [4]. The idea behind regulated competition in health insurance is that insured persons who are not satisfied with the premium or quality of care provided, or just can get a better deal, will opt for a different insurer. This would force insurers to strive for good prices and quality of care for their insured. It does not imply that all insured who are dissatisfied should switch to another insurer nor that only dissatisfied people switch. According to Hirschman, the mechanism will work best when some insured go to another insurer, thus showing their dissatisfaction, while others remain with this insurer, thus providing the resources for the insurer to improve [12]. Still, switching will only influence both price and quality if there are indeed differences between insurers and if the insured base their choice on these aspects [8]. For collective contracts it might be that insured simply choose a collective contract without considering the price-quality balance. Therefore, it is important to examine which people tend to switch more and what their reasons are for doing so.

There are indeed differences in premium. Information about this is easily accessible on the internet (e.g. [13]). Differences in quality of care for the insured of different insurance organizations are much less clear if they exist at all. Recently, the Dutch Healthcare Authority (NZa) reported that there are hardly any differences between the basic packages from different insurers, but there are difference in complementary insurance [14]. For the relatively young and healthy, quality aspects are less important than premium. We therefore expect relatively young and healthy people to switch more often than people who are older and/or in less good health. The latter are also more dependent on their insurer and might not switch because they know what they have, but do not know what they will get when going to another insurance company.

Relatively young and healthy people will base their choice of insurance plan probably on considerations like premium or paperwork related to switching insurance company, considerations that are unrelated to quality of care. This was found in several European countries, including the Dutch situation before the recent insurance reform [15-18]. Only people who frequently use health care, such as the chronically ill and disabled, have substantial experience with health care to base their choice on considerations that are related to the quality of health care. It is only these groups that can provide insurance companies a signal related to the quality of health care [19]. Thus, a difference in reasons for switching can be expected between those who use health care frequently, and those who do not use health care on a frequent basis.

An interesting aspect of the new Dutch health insurance system is the possibility of collective insurance contracts [20]. Any group of people, e.g. united through their work place, a sports association or patient organization, can take out insurance at a discount of maximum 10% on the basic insurance package if an insurance company is interested to offer a collective contract. Insurance companies are not allowed to base discounts on the relative risk of the people for whom the collective offer is available. The size of the discount can only depend on the size of the collective. Collectives are interesting for (at least) two reasons. First of all, access could be (unintentionally) easier for some people, such as those with a job which are mainly people in good health. And secondly, for those who have access to collective insurance, the choice situation is less complex. Information overload might lead to stress [21]. If the number of health plans to choose from is too large, people are less inclined to switch because of increased costs of collecting information and coming to a decision [17].

In this article we compare actual switching from insurer and stated reasons for switching in the general population and a specific group of insured: the chronically ill and disabled.

In this article two questions will be answered:

1. How many insured switch insurance company in the new system and are there differences between population categories, defined in socio-demographic and health characteristics?

2. What are reasons and barriers for switching? What is important in choosing an insurance package? Do these reasons and barriers differ according to socio-demographic and health characteristics?

We hypothesize that:

• Young and healthy people are more inclined to switch insurer than elderly and chronically ill or disabled people.

• The chronically ill and disabled are less often insured via a collective contract.

• People who do not often use health care, healthy insured, attach more importance to the level of the premium, while people who use health care frequently, chronically ill or disabled, attach more importance to the content of the insurance package.

• Being able to join a collective is an important reason for switching but less often for the chronically ill and disabled.

## Methods

Questions on switching health insurer and insurance plan and reasons for doing so were asked to the Dutch Health Care Consumer Panel, a cross-section of the Dutch population (N = 1516) and the National Panel of the Chronically ill and Disabled (N = 3800) in April 2006.

### Dutch Health Care Consumer Panel

The Dutch Health Care Consumer Panel consists of about 1500 people and is representative for the general population for age and sex. Members of the panel receive a questionnaire four times a year. Members can quit the panel any time. Every two years, one third of the panel members is renewed. This renewal ensures that the panel remains a cross-section of the population, that members do not develop specific knowledge of and attention for health care issues and no "questionnaire-fatigue" occurs. New members for the panel are sampled from the general population. Sampled people receive an information letter about the panel and are called within a week after receiving that letter. If they are interested they receive first a questionnaire on background characteristics. When that questionnaire is returned they are considered members of the panel.

### National Panel of Chronically ill and Disabled

The National Panel of Chronically ill and Disabled (NPCD) is a nationwide data collection and research programme investigating the need and use of care and living circumstances of chronically ill and disabled people (15 years and older) in the Netherlands [22]. Chronically ill patients were recruited via a representative sample of 29 general practices in order to have medically assessed diagnoses. Disabled people were selected through a screener question on disabilities in two large-scale regular population surveys. Panel members take part into the panel during four years. The NPCD is representative for the population of independently living people of 15 years and older with a (somatic) chronic illness or disability. All data are standardized to a standard population based on the proportion between the number of chronically ill people and the number of disabled people in the Dutch population.

### Data collection

In April 2006 postal questionnaires were sent to 1516 members of the Dutch Health Care Consumer Panel and to 3757 members of the National Panel of the Chronically ill and Disabled. Among other things, members of both panels were asked about their choices for a health insurer and insurance plan and the reasons for their choice. The questionnaire was returned by 1198 members of the Dutch Health Care Consumer Panel (response 79%) and by 3211 members of the National Panel of the Chronically ill and Disabled (response 86%). The protection of the collected data from both panels was laid down in privacy regulations, safeguarding ethical consent, and registered by the Dutch Data Protection Authority (nr. 1262949 and nr. 1283171).

### Analyses

As the chronically ill and disabled are older than a sample from the general population, the panels used for this article are not comparable on age, sex and education (Table 2). A weight factor for the chronically ill and disabled was used to make both panels comparable on these characteristics. A total of 18 weight factors were used for the combination of age (18–44; 45–65; 65 and older), sex (male; female) and education (lower; intermediate; higher). For two reasons both the unweighted and weighted results will be presented. First, weighting influences conclusions being drawn. Differences in switching behaviour and reasons might not be related to chronic illness or disability but to socio-demographic composition. Second, although differences between both populations might primarily be determined by characteristics like age, sex and education the fact remains that the population of the chronically ill and disabled is older, has a higher percentage of women and is lower educated, compared to the general population. Cross tables and logistic regression were used to test the hypotheses. For these analyses we used the statistical program SPSS version 14.0.

**Table 2:** Description of respondents: age, sex and education, April 2006

	*Chronically ill and disabled (n = 3211)*	*General population (n = 1198)*
**Age**		
18–44	14%	34%
45–64	41%	42%
65 →	46%	24%
**Sex**		
Male	36%	44%
**Education**		
Lower	46%	19%
Intermediate	39%	72%
Higher	15%	9%

## Results

### Switching health insurer

Unweighted figures show that 14% of the chronically ill and disabled switched insurer at the introduction of the health insurance reform (Table 3). In the general population the percentage of switchers was 21%. However, the magnitude of the difference is strongly influenced by the fact that the chronically ill and disabled are in general older, more often female and less highly educated than the general population. The weighted percentage for the chronically ill and disabled is 19%.

**Table 3:** Percentage of people switching health insurer and their characteristics, weighted for age, sex and education, unweighted figures between brackets, April 2006

	*Chronically ill and disabled*	*General population*
**Age**		
18–44	24% (24%)	27%
45–64	18% (17%)	20%
65 →	12% (10%)	13%
**Sex**		
Male	15% (14%)	18%
Female	21% (16%)	23%
**Education**		
Lower	11% (10%)	14%
Intermediate	21% (18%)	22%
Higher	25% (23%)	23%
**Perceived health status**		
Bad	15% (12%)	21%
Good	20% (17%)	20%
Very good	29% (25%)	23%
**TOTAL**	19% (14%)	21%

The percentage of switchers in 2006 is much higher than in the situation before the insurance reform, when it was approximately 3% for the publicly insured and 6% for the privately insured [23,24].

Results show that younger people are switching more often than older people. 27% of the 18–44 year old switched insurance company (Table 3). This pattern is the same for the general population and the chronically ill. Although people 65 years and older are switching less often than younger people, they still switch more often than people did in the old system. Men have switched less often than women. 18% of the male population and 23% of the female population switched their insurance company. This pattern is again the same for the general population and the chronically ill and disabled. Higher educated people switched more often than lower educated people. This might be caused by differences in knowledge and understanding of information.

**Figure 1:**
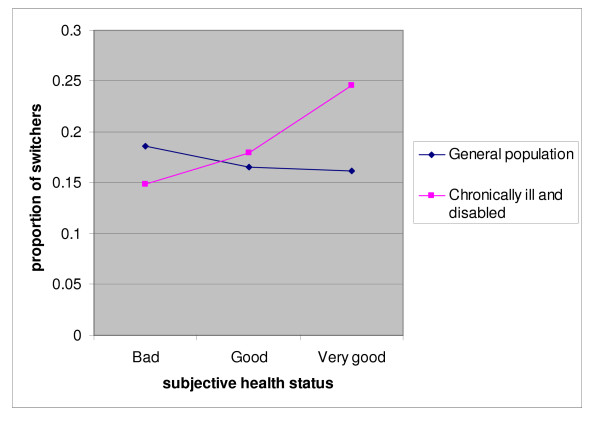
Switching behaviour and subjective health status for the general population (n = 1086) and the chronically ill and disabled (n = 3132); April 2006.

Healthier people more often switch health insurance (Table 4). However, there is an interesting interaction. In the general population people with very bad self-perceived health switch more often than the same category amongst the chronically ill and disabled. For those with very good health the opposite is the case: there are more switchers amongst the chronically ill. Figure 1 shows the difference in effects of subjective health status between the general population and the chronically ill on their switching behaviour. It might be that people from the general population who perceive their health as very bad are in better health than people from the chronically ill and disabled who perceive their health as very bad. For the chronically ill and disabled population who perceive their health status as very good, it can be reasoned that they very well know what they need insurance for and that they feel capable to search for the most appropriate health insurer and insurance plan.

**Table 4:** Logistic regression model for switching health insurer, April 2006

	Exp (B)	p-waarde
**Age**		
18–44	1.96	0.00
45–64	1.71	0.00
65 →	reference	
**Sex**		
Male	0.79	0.04
Female	reference	
**Education**		
Lower	0.44	0.00
Intermediate	0.73	0.03
Higher	reference	
**Perceived health status**		
Bad	0.54	0.00
Good	0.67	0.04
Very good	reference	
**Panel**		
Dutch Health Care Consumer Panel	0.59	0.20
National Panel of Chronically ill and Disabled	reference	
**Interactions with panel**		
**Age**		
18–44	1.11	0.72
45–64	0.94	0.80
**Sex**		
Male	1.07	0.73
**Education**		
Lower	1.32	0.44
Intermediate	1.25	0.46
**Perceived health status**		
Bad	2.22	0.00
Good	1.53	0.10
**Constant**	0.33	0.00

The first hypothesis is partly confirmed. Young and healthy people indeed switch insurer more often than elderly or people in bad health. It was not confirmed that the chronically ill and disabled switch less often than the general population when both populations are comparable on age, sex and education. However, given the fact that these two populations do differ on these variables we have to take into account that the actual percentage of switchers is lower in the population of chronically ill and disabled, as shown by the unweighted data.

Many people have used the possibility to obtain a discount on the premium for the basic package by joining a collective. In the general population 56% did so and among the chronically ill and disabled 42% unweighted and 47% when the data are weighted towards the age, sex and educational distribution of the general population (not presented in tables). The logistic regression (not presented in tables) shows no significant differences between both groups when additionally controlling for subjective health. People in working age, of male gender, higher educated and in better subjective health more often choose for a collective insurance. This is related to the fact that most of the collective discounts were offered via people's job [25].

So, in a multivariate analysis the chronically ill and disabled are not less often using collective insurance, but given the fact that they differ systematically from the general population in their socio-demographic characteristics and subjective health the unweighted percentage of collectively insured is lower among the chronically ill and disabled. However, this seems not to be attributable to the mere fact that they are chronically ill or disabled. The second hypothesis is therefore refuted.

### Reasons for switching and importance of different aspects in choosing an insurance package

Collective offers are reported most often as reason for switching health insurer, for both the chronically ill and disabled and the general population (Table 5). The percentage of the chronically ill and disabled that reports content of the insurance package, both coverage in general and coverage of compulsory insurance, as reasons for switching is much higher than it is for the general population. Quality of care was not often a reason to switch, neither for the chronically ill nor for the general population.

**Table 5:** Reasons for switching health insurer (data for the chronically ill and disabled weighted to the age, sex and educational composition of the general population, unweighted figures between brackets); April 2006

	Chronically ill and disabled (N = 544)*	General population (N = 222)*
Attractive collective offer	68% (66%)	56%
Premium of the package offered	38% (38%)	41%
Coverage of the package offered	33% (33%)	15%
Coverage compulsory insurance	25% (23%)	18%
Premium compulsory insurance	15% (16%)	19%
Other reason	10% (11%)	11%
Service level of the insurer	10% (9%)	9%
Coverage basic package	8% (8%)	6%
Quality of care	6% (5%)	5%
In kind/restitution policy	4% (4%)	3%
Deductible in combination with discount	2% (3%)	5%

* number of switchers

Besides reasons for switching we asked respondents how important different aspects, like premium and content of the package, are in choosing an insurance package. Table 6 shows the percentage of people who switched insurer that rate an aspect as (very) important. This analysis shows differences between the general population and the chronically ill for quality, free choice of health care provider, free acceptance for compulsory insurance and the coverage of specific devices and medications. For each of these items the percentage of switchers amongst the chronically ill that rate the item as important is higher than it is for the general population. The chronically ill and disabled seem to be more interested in content related issues than insured from the general population.

**Table 6:** Percentage of switchers and non-switchers who rate an aspect as (very) important (weighted figures, unweighted figures between brackets)

	Chronically ill and disabled		General population	
	Switchers	Non-switchers	Switchers	Non-switchers

Premium	86% (84%)	73% (73%)**	83%	75%***
Coverage	94% (93%)	92% (92%)	92%	94%
Premium compulsory insurance	86% (83%)	76% (76%)**	84%	77%***
Coverage compulsory insurance	90% (89%)	88% (88%)	91%	89%
Deductible in combination with a discount	18% (21%)	28% (31%)**	20%	19%
Quality of care	89% (87%)	91% (91%)	80%*	89%***
Service of insurer	86% (86%)	89% (90%)	82%	88%***
In kind/restitution policy	41% (45%)	48% (51%)**	42%	46%
Collective offer	72% (73%)	51% (51%)**	70%	50%***
Free choice of health care provider	77% (76%)	60% (62%)**	60%*	56%
Free acceptance for compulsory insurance	79% (78%)	66% (68%)**	70%	65%
Content like specific health arrangements	38% (40%)	39% (44%)	33%	37%
Coverage of specific devices of medications	73% (74%)	73% (75%)	55%*	62%***

* significant difference between general population and chronically ill and disabled switchers

** chronically ill and disabled: significant difference between switchers and non-switchers

*** general population: significant difference between switchers and non-switchers

The third hypothesis is confirmed. For the general population, people who generally do not use health care often, premium is more important than content, while the chronically ill and disabled value content of the insurance package as well. However, quality of care is not important for either group as a reason for switching (Table 5). Finally, the fourth hypothesis, stating that a collective offer is less often an important reason for switching for the chronically ill and disabled was refuted. For both populations switching health insurer is related to collective offers, and being chronically ill or disabled has not been found to be related to switching behaviour.

## Discussion

In this article we have studied switching of health plan by Dutch insured after the major insurance reform of January 2006. We have both looked at actual switching and at reasons for doing so. We have compared the general population with people with a chronic illness or disability.

The percentage that switched health insurer at the introduction of the new insurance system was high compared to the situation in the old system. The chronically ill and disabled switched health insurer less often than the general population. However, when we take into account the fact that the chronically ill and disabled are older, more often female and less highly educated than the general population, the difference disappears. Switching was not based on considerations of quality of care for either group. Premium and collective offers were more important for mobility of insured in the general population. In line with our expectations, content of insurance package was more important for the chronically ill and disabled than for the general population.

Collective offers are important reasons for switching and many people have chosen a collective insurance. The percentage covered by a collective insurance is higher among the general population, but again, this is explained by the differences in socio-demographic composition.

The philosophy behind introducing more choice in health care systems is that it is a way to protect the well-being of insured [19]. Switching insurer is seen as an important signal to insurance companies that insured are dissatisfied with levels of service or quality of care. However, quality of care only plays a role when there are indeed differences between insurers in this respect and when people who switch act in these differences in quality. Neither seem to be the case. People do not perceive differences between health insurers in quality of care [26] and quality of care was mentioned as a reason to switch by only a small percentage of both groups. If reasons for switching are unrelated to the quality of care, it is hard to believe that switching influences the quality of care.

Mobility of insured people is an important pillar in the new Dutch insurance system. We studied two groups of insured: the general population and the chronically ill and disabled. The chronically ill and disabled have substantial experience with health care and are able to judge service level and quality. These groups therefore have the possibility to give insurance companies a signal that they are dissatisfied with these aspects. The signals healthy people give mainly relate to the premium. In order to use mobility of insured as a mechanism to enhance service and quality, it is important that chronically ill and disabled switch insurance company when they are dissatisfied. In the new Dutch system barriers to switch insurance company have been removed in order to let the insured influence premium, service and quality of care by switching health insurer. As yet there are no signs of barriers to switch insurer for the chronically ill and disabled [27].

It is unclear what the percentage of people switching insurers should be in order to create competition between insurance companies on premium and quality. It might be that the mere threat of mobility of insured is enough to keep insurance companies active. However, high levels of mobility could also have negative effects; mobility brings administrative costs and when consumer mobility is high collective costs will increase. These collective costs could be the cause of increased premiums.

Switching insurer was rather high in this first year of the new insurance system. Mass media attention might have made choice situation more salient. Furthermore, in the old system people with private insurance could not easily switch insurer. In the first year of the new system, insurance companies were generous in accepting people for additional insurance. There were hardly any problems with taking out additional insurance. People might have taken the opportunity to switch insurance company or to take out a large additional insurance package. It could be questioned whether the increased mobility will continue in the future, or that it was just a onetime event. Latest figures suggest that it was indeed a onetime event, switching rates have decreased to the level of before the introduction of the new insurance system [28].

In the Dutch system, insurers must accept every applicant for the basic package, irrespective their age, health status and other characteristics. For additional insurance there are no such rules; insurers can refuse applicants for additional insurance. Although insured are allowed to have their basic insurance from another insurer than their additional insurance, there is a possibility of risk selection through the additional insurance. It is very unlikely that insurers will remain generous in accepting people for additional insurance. Insured can be locked in with their insurer, because of their additional insurance. Although possible, it is not likely that they switch for the basic insurance only, because it is administratively unattractive. It will be interesting to examine whether this will happen in the future. In more general terms: do all insured (feel to) have the possibility to switch to another insurer?

In this article the general population was compared to the chronically ill and disabled. This comparison was based on the idea that the general population on average use less care than the chronically ill and disabled. Therefore, for the chronically ill and disabled content of the insurance package is more important. Besides, it is probably easier to know what kind of care is needed when someone is chronically ill or disabled, than when it is unknown what kind of illness one might be confronted with in the future. The chronically ill and disabled know what kind of care they probably need for their illness and therefore know what they need insurance for. Differences between the chronically ill and disabled and the general population are small when the difference in composition between both categories is taken into account. It is not disablement or chronic illness as such that keeps people from switching health plans, but rather the fact that older people and lower educated people tend to switch less often and are over-represented among the chronically ill and disabled. Irrespective of how one interprets this result, the fact remains that some population categories switch less often. This can be seen as a sign of the existence of inequalities. If these differences are related to specific groups that can be characterised according to needs, like certain patient groups, we speak of inequities [29]. Therefore, how benefits of the new system of regulated competition are distributed and why this is so should be investigated [9].

The transition from a planned health care system to regulated competition is an example of a market transition in a specific area within a market economy. Market transitions have been claimed to increase inequalities [10,11]. In this case inequalities could show up in different fields. In the insurance market, some insurance organizations gain and others loose and some categories of the population might profit more from competition than others. In the health care provision market, some providers might be better able to adapt to a competitive environment. And finally, in the health care purchasing market some providers might be able to negotiate attractive contracts with insurance organizations and others not. Market transition theory focuses attention to the possibility of developing inequalities that might result in inequities. Even though the new Dutch insurance system has guarantees to prevent cream skimming inequities could arise.

## Conclusion

There is increased mobility in the new system for both the general population and the chronically ill and disabled. This however is not based on quality of care. The general assumption of the system is that mobility between insurers is based on considerations of both premium and quality. Thus, consumers would exert pressure towards insurers to keep premium low and improve quality of care. If reasons for switching are unrelated to the quality of care, it is hard to believe that switching influences the quality of care. The Dutch system is designed to prevent the development of inequalities between for instance healthy and unhealthy people, such as the chronically ill and disabled. As yet there are no signs of barriers to switch insurer for the chronically ill and disabled. This however could change in the future and it is therefore important to monitor changes.

## Competing interests

The author(s) declare that they have no competing interests.

## Authors' contributions

JdJ drafted the manuscript, performed the statistical analyses, and contributed to all other aspects of the study. AvdB contributed to the acquisition of the data and was involved in drafting the manuscript. PG contributed to the acquisition of the data, drafting the manuscript and critical revision of this manuscript. All authors have given final approval of the submitted manuscript.

## Pre-publication history

The pre-publication history for this paper can be accessed here:


